# Supplementary material for: Repeatability of flatfish reflex impairment assessments based on video recordings
Source: PLoS One. 2020 Feb 26;15(2):e0229456. doi: 10.1371/journal.pone.0229456 (PMC7043772; doi:10.1371/journal.pone.0229456)
Supplement: Attachment — Submitted filename: reply_to_editor.docx [file pone.0229456.s001.docx]

Ostend, January 27, 2020

Dear Dr Hewitt,

Please find attached a revised version of our manuscript entitled “Repeatability of flatfish reflex impairment assessments based on video recordings”. We have addressed all of the very useful comments and revised the manuscript accordingly. To make the changes clear, we have provided details on a point-by-point basis below.

Thank you for your consideration.

On behalf of my co-authors, kind regards,

**Journal requirement: naming of files**

As requested we have paid attention to the file and formatting style and naming requirements as outlined in the provided guides for authors documents. Accordingly, adjustments to the title page were made. Please note that the formatting of the affiliations are inconsistent in the example provided (i.e., there seem to be a space missing between superscript and affiliation address in some instances, check ^1^ Department, Institution, City, State, Country vs ^2^).

**Journal requirement: figure 1 – license to publish**

Although all persons pictured were facing with their backs to the camera and the purpose of this image is to illustrate the projection of the video clips onto a big screen and not to show the audience or individual participants, we have blurred the audience in photoshop to make sure that no one is identifiable and added this as a comment to its figure caption (L123-L124).

**Reviewer #1:**

**Comment 1 – Weird spacing in the intro.** We have corrected the weird spacing and apologise for it. It was an artefact from sharing a manuscript draft between North American and European Microsoft Office Word packages, which caused compatibility issues with the formatting, and eventually I created a new version by copying and pasting.

**2 – Additional information needed in intro.** As requested, we included a short paragraph at the beginning of the introduction outlining the concept of reflex scoring and why it is used (L47-L55). Note that the numbering of in-text citations has changed to accommodate for extra referencing of studies.

**3 – Link between hypoxia and impairment.** See comment 2 above. This paragraph also mentions how the exposure to air from hypoxia could contribute to impaired reflex responsiveness (L53-L55).

**4 – Video/crowd sourcing idea**. We thank the reviewer for this appreciation of our efforts. It all started off as a ‘pet project’, and soon we realized that we could make a very useful contribution to the study of reflex responsiveness of fish and experimental design in fisheries science in general.

5 – **Table 7**. We are not sure whether the reviewer refers to Table 1 here, because there is no Table 7? We have added another reference to the caption of Table 1 (L153).

**6 – Selection of clips.** We have added a sentence to explain how clips were selected and added a histogram of the distribution of averaged ‘silver standard’ scores (Fig 3). These were based on scores by three experts, who were experienced raters who were involved in developing the methodology and who scored 12 unmodified, original clips in a separate video. These 12 clips were then used to create the scoring video by introducing duplicates, and falsifying air exposure information. We also modified Figure 4 (now Figure 5), to indicate how the percentile ranges of pooled raters (by experience) compared to the ‘silver standard’ scores averaged across the three expert raters (L305-L307).

**7 – Choice of fictive air exposure periods.** These air exposure periods were chosen for two reasons: to represent commercial fishing practices (depending on catch volumes which can be large), it may take at least 15 min to 30 min until all air-exposed catch has been sorted by the crew. Secondly, the longer air exposure period was chosen to increase any expectation potential (L187-L189).

**8 – Choice of reflexes.** If the reviewer refers to what is called “head complex” or “operculum” in the literature and scores the functioning of the breathing apparatus, then this reflex was not selected, because it was considered that it may be difficult to discern for a rater whether the gills/operculum and/or mouth of a sole would be moving from a video clip.

**9 – Veterinarians in the study.** In the material and Methods section, we have specified that experience can refer to having assessed any vertebrate animal for reflex behaviours (L212-L213), and mentioned in the Results section how many of the raters with some experience have had experience with scoring behavioural responses among vertebrate animals other than fish (L235-L236). None of the practicing veterinarians had indicated any prior experience with scoring reflexes.

**10 – Visualize some of the data.** We suggest to include as an online supplement a video which we produced that includes a powerpoint lecture, training video and commentary. Other than that, we think that the structure and experimental design was sufficiently and succinctly summarized in the tables we provided. But we also included another histogram to illustrate the distribution of ‘silver standard’ scores (see comment 6).

**11 – Superficial discussion.** In places, we have revised the discussion to relate our results in greater depth to recent and relevant literature (L381-L383; L404-L406).

**12 – Additional table or paragraph outlining general recommendations.** We think that our concluding and several other paragraphs in the discussion list our recommendations, and we do not see the necessity to add another table to keep this article more concise.
